# Multi-community effects of organic and conventional farming practices in vineyards

**DOI:** 10.1038/s41598-021-91095-5

**Published:** 2021-06-07

**Authors:** Noémie Ostandie, Brice Giffard, Olivier Bonnard, Benjamin Joubard, Sylvie Richart-Cervera, Denis Thiéry, Adrien Rusch

**Affiliations:** INRAE, Bordeaux Sciences Agro, ISVV, SAVE, F-33140 Villenave d’Ornon, France

**Keywords:** Agroecology, Biodiversity

## Abstract

Understanding the response of biodiversity to organic farming is crucial to design more sustainable agriculture. While it is known that organic farming benefits biodiversity on average, large variability in the effects of this farming system exists. Moreover, it is not clear how different practices modulate the performance of organic farming for biodiversity conservation. In this study, we investigated how the abundance and taxonomic richness of multiple species groups responds to certified organic farming and conventional farming in vineyards. Our analyses revealed that farming practices at the field scale are more important drivers of community abundance than landscape context. Organic farming enhanced the abundances of springtails (+ 31.6%) and spiders (+ 84%), had detrimental effects on pollinator abundance (− 11.6%) and soil microbial biomass (− 9.1%), and did not affect the abundance of ground beetles, mites or microarthropods. Farming practices like tillage regime, insecticide use and soil copper content drove most of the detected effects of farming system on biodiversity. Our study revealed varying effects of organic farming on biodiversity and clearly indicates the need to consider farming practices to understand the effects of farming systems on farmland biodiversity.

## Introduction

Agriculture, which dominates more than one third of the world’s terrestrial surface, is recognized as one of the main drivers of biodiversity loss^1^. The growing demand for agricultural commodities is expected to strengthen the expansion and intensification of agricultural land, with strong impacts on biodiversity ^2,3^. To overcome this challenge, the land-sharing approach suggests promoting ecosystem services delivered by biodiversity through the development of more environmentally friendly agriculture supporting both production and biodiversity conservation on the same land ^4,5^. Organic farming, which is often seen as a prototype of such agriculture, is expanding, and approximately 71 million hectares of farmland are currently under certified organic farming at the global scale ^6,7^. Promoting organic farming is one of the main agro-environmental policies around the world ^8^. For instance, in its recent Green Deal, Europe Union officially targets to reach 25% of its total farmland under organic farming by 2030. However, the benefits of certified organic farming in reducing the environmental footprint of agriculture are widely debated, and large uncertainty exists around the performance of such farming ^9,10^.

Overall, biodiversity is known to benefit from organic farming ^11–13^. Several meta-analysis have found that organic farming increases the abundance of organisms by 50% and species richness by 30%^12,13^. Organic farming is particularly significant and beneficial to soil microbes, plants, pollinators or predators ^14,15^. However, the claim that agri-environment measure such as organic farming contribute to halting the biodiversity decline has been recently challenged ^16,17^. Several studies have reported that the effects of organic farming are highly variable, and recent evidence even pinpointed that organic farming could have negative effects on some biodiversity components ^13,17–19^. Birkhofer et al. (2014) reported that there are both winners and losers of organic farming across a large range of organisms including bird, ground beetle, spider, butterfly and moth communities. Examining how multiple species or functional groups respond to organic farming is of major importance to understand the actual effects of this popular agri-environment measure on biodiversity.

The beneficial effects of certified organic farming on biodiversity are usually attributed to the ban of synthetic pesticides and fertilizers as well as to higher levels of soil organic matter or longer and more diversified crop rotations compared to those of conventional farming ^20,21^. However, several practices allowed by organic farming certification standards such as copper or sulphur-based fungicides, microbial insecticide or intensive soil tillage have negative impacts on biodiversity ^22,23^. Moreover, studies explicitly considering the different farming practices actually applied in fields when comparing the relative effects of organic and non-organic management are rare. It is likely that this poorly-explored source of variability might partly explain some inconsistent results reported in the literature. In addition, it has been recently argued that simplifying technical choices of farmers into broad comparisons of conventional versus alternative systems could limit our understanding of the socioecological impacts of agriculture and could precludes the development of novel systems that can potentially deliver multiple beneficial outcomes ^24^. Explicitly analyzing the impacts of farming practices on multitrophic biodiversity is therefore a necessary step to understand the variable performance of organic farming and to set the scene for the ecological intensification of farming systems.

A considerable number of species have large home ranges and exploit multiple resources in different habitats, leading to a major effect of landscape context on species assemblages in agricultural fields ^25,26^. Despite its local effects on biodiversity, organic farming at larger spatial scales may therefore affect biodiversity dynamics in agricultural landscapes ^27,28^. Recent evidence have demonstrated that the effects of organic farming on biodiversity and ecosystem services are scale dependent ^28,29^. However, only few studies have considered how the spatial expansion of organic farming affect biodiversity.

The aim of this study was to quantify the impacts of organic farming and underlying farming practices on vineyard biodiversity considering seven different taxonomic groups, from microbes to pollinators. In addition, we investigated the scale of the effects of organic farming on these different groups, from the field to the landscape scale. We decided to focus on vineyard-dominated landscapes of southwestern France as vineyards of this region can be intensively managed (on average 17 pesticide treatments are applied per unit area each year, ^30^ and because organic farming area is rapidly increasing (area under organic farming increased by 300% in ten years ^31^. We expected an overall positive effect of organic farming on the abundance and taxonomic richness of belowground and aboveground communities. Moreover, we hypothesized that the scale of organic farming effects would depend on the community considered, with a priori expectations of a stronger effect of local management on decomposer communities (with limited dispersal abilities) and greater importance of organic farming proportion in the landscape for pollinator and predator communities (with higher dispersal ability).

## Results

### Effect of organic farming at the field and landscape levels

**Figure 1:**
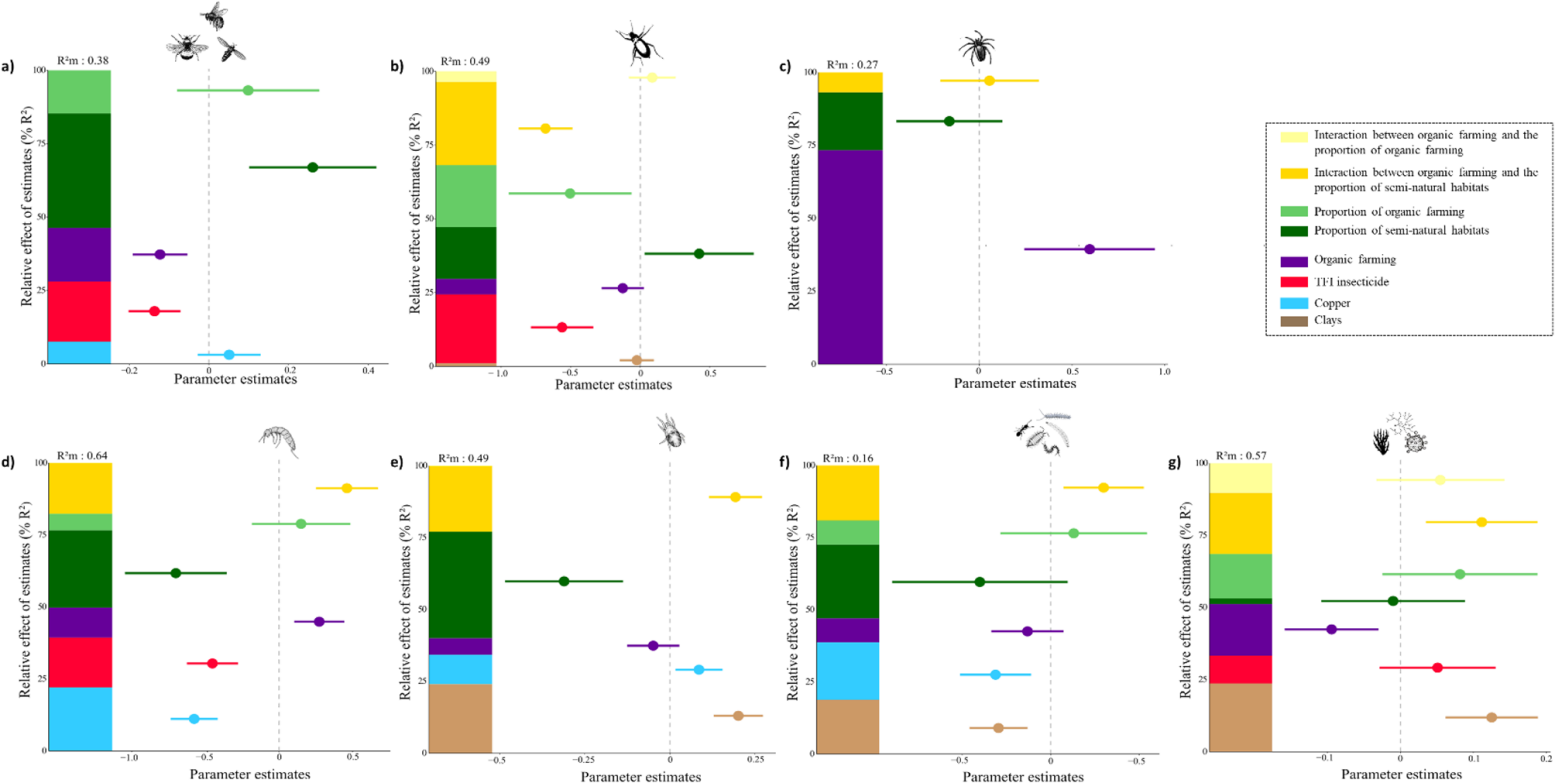
Results of the best models explaining the abundances of **(a)** pollinators, **(b)** ground beetles, **(c)** spiders, **(d)** springtails, **(e)** mites, **(f)** soil microarthropods and **(g)** microbial biomass according to the type of farming system (organic or conventional), landscape context, farming practices independent of farming systems and soil characteristics. Stacked bars show the relative effects of estimates (%R^2^) for each explanatory variable calculated as the ratios between the parameter estimates and the sum of all parameter estimates based on a model averaging approach applied to model 1. Points are estimates of the model coefficients, and lines represent confidence intervals. All continuous predictors were scaled to interpret parameter estimates at comparable scales. All individual plots representing the effects of significant explanatory variables of Fig. 1 are provided as supplementary material (Fig S1-S8). Note that results of best models for models 2 explaining abundances of the different groups by specific farming practices, landscape context and soil characteristics are provided in Figure S10. This figure was made using R version 4.0.3 (https://www.R-project.org/) and Inkscape 1.0 (www.inkscape.org).

Organic farming at the field scale was retained in all best models (models with a ΔAICc < 2 in the model averaging procedure) explaining the abundances of above- and belowground communities but had contrasting effects on these communities (Fig. 1, all plots representing the effect of significant explanatory variables on each response variables are provided as supplementary material Figure S1-S8, see also Table S1 for coefficient estimates in all best models). Organic farming, in comparison to conventional farming, increased the abundance of spiders (+ 84%) and springtails (+ 31.6%) but decreased the abundance of pollinators (− 11.6%) as well as soil microbial biomass (− 9.1%) (Suppl. Mat. Figure S1). Organic farming at the field scale was the most important variable explaining spider abundance, as it accounted for 73% of the variance explained by the model (variance explained by the fixed effects, R^2^m, was 27%), while it explained 18% of the explained variance for the abundance of pollinators (R^2^m = 38%), 17% of the explained variance for soil microbial biomass (R^2^m = 57%) and 10% of the explained variance for the abundance of springtails (R^2^m = 64%) (Fig. 1). Organic farming had no effects on the abundances of ground beetles, mites and soil microarthropods (Fig. 1). Organic farming was less important in explaining changes in taxonomic richness of the different communities, as it only had a strong and positive effect on spider species richness (76% of explained variance; R^2^m = 26%; see Figure S9). At the landscape scale, increasing the proportion of organic farming decreased the abundance (accounting for 20% of explained variance, R^2^m = 49%) and taxonomic richness of ground beetles (accounting for 27% of explained variance, R^2^m = 55%) as well as the abundance of soil microarthropods (accounting for 37% of explained variance, R^2^m = 16%) (Fig. 1, Suppl. Mat. Figures S3 and S9).

Our analyses revealed that tillage intensity mainly mediated the observed field-scale effects of organic farming on the above- and belowground communities (model 2 outputs in Suppl. Mat. Figure S10). Tillage intensity decreased the abundances of pollinators and ground beetles as well as microbial biomass, while it increased the abundance of springtails (Suppl. Mat. Figure S2). No significant effect of tillage intensity was found on the taxonomic richness of the above- and belowground communities (model 2 outputs in Suppl. Mat. Figure S11). Our analyses also revealed a significant interaction effect of tillage intensity at the field scale and the proportion of organic farming in the landscape on the abundance of soil microarthropods (model 2 outputs in Suppl. Mat. Figure S10). Fields with higher tillage intensity had a higher abundance of soil microarthropods than fields with lower tillage intensity in landscapes with a high proportion of organic farming, while the opposite was true in landscapes with a low proportion of organic farming.

### Effect of semi-natural habitats at the landscape scale and interaction with the local farming system

The proportion of semi-natural habitats was selected in all the best models but had opposite effects on the abundance or biomass of the above- and belowground communities (Fig. 1). Increasing the proportion of semi-natural habitats enhanced the abundance of pollinators (38% of explained variance, R^2^m = 38%) and ground beetles (17% of explained variance, R^2^m = 49%), while it decreased the abundance of springtails (26% of explained variance, R^2^m = 64%) and mites (37% of explained variance, R^2^m = 49%) (Suppl. Mat. Figure S4). The proportion of semi-natural habitats never affected the taxonomic richness of the above- and belowground communities (model 1 and 2 outputs in Suppl. Mat. and Figures S9 and S11).

The interaction between the local farming system and the proportion of semi-natural habitats was selected in all best models explaining the abundance or biomass of the above- and belowground communities, except for the pollinator community. Fields under organic farming had a higher abundance of ground beetles than conventional fields when located in landscapes with a low proportion of semi-natural habitats, while the opposite was true in landscapes with a high proportion of semi-natural habitats (Suppl. Mat. Figure S5). In contrast, fields under organic farming had lower abundances of springtails, mites, and microarthropods as well as microbial soil biomass than conventional fields in landscapes with a low proportion of semi-natural habitats, while the opposite was true in more complex landscapes with a high proportion of semi-natural habitats (Suppl. Mat. Figure S5).

### Insecticide use

Independently of the type of farming system, insecticide use intensity decreased the abundances of pollinators (20% of explained variance, R^2^m = 38%), ground beetles (23% of explained variance, R^2^m = 49%) and springtails (17% of explained variance, R^2^m = 64%) as well as the taxonomic richness of pollinators (67% of explained variance, R^2^m = 19%) (Fig. 1, Suppl. Mat. Figures S6 and S9). No significant effects of insecticide use intensity were found on the taxonomic richness of the other above- or belowground communities.

### Soil copper and soil texture

The amount of copper in the soil was found to affect belowground communities as it increased the abundance of mites (10% of explained variance, R^2^m = 49%) but decreased the abundances of springtails (22% of explained variance, R^2^m = 64%) and other microarthropods (19% of explained variance, R^2^m = 16%) (Fig. 1, Suppl. Mat. Figure S7). The proportion of clay in the soil was an important covariable for belowground communities, as its increase was associated with increases in the abundance of mites and soil microbial biomass but a decrease in the abundance of microarthropods (Fig. 1, Suppl. Mat. Figure S8). The taxonomic richness of ground beetles was negatively affected by the proportion of clay in the soil, while the taxonomic richness of microarthropods benefited from an increase in clay content (Suppl. Mat. Figures S8 and S9).

## Discussion

Our study reveals contrasting effects of organic and conventional farming on biodiversity across multiple trophic groups, with strong effects on abundance and limited effects on taxonomic richness of the different groups considered. Explanatory power of models indicate that the effects of farming practices were better captured on community abundances than on taxonomic richness as explanatory power of models fitted on taxonomic richness were low, except for carabids (Figure S9 and see below). Among the seven groups studied, organic farming at the field scale enhanced the abundances of springtails (+ 31.6%) and spiders (+ 84%), had detrimental effects on pollinator abundance (- 11.6%) and soil microbial biomass (- 9.1%), and did not affect the abundance of ground beetles, mites or microarthropods. Using a multiscale design that made it possible to evaluate the scale of the effects of organic farming on the seven taxonomic groups considered, we found that organic farming at the field scale is a more important driver of above- and belowground community characteristics than the proportion of organic farming at the landscape scale. At the field scale, we show that beyond the type of farming system, features such as tillage intensity, insecticide use and soil copper content are important variables that affect biodiversity, with a predominance of negative impacts on the abundances of these communities.

Previous studies have demonstrated that, on average, organic farming increases taxonomic richness by approximately 30% and abundance by 50% but have also reported highly variable responses among taxa ^11–13^. Our study based on large sampling of multiple communities operating at different trophic levels highlights the strong variability in the response of different trophic groups to organic farming. We therefore did not validate our initial hypothesis about an overall positive effect of organic farming on the abundance and taxonomic richness of multiple communities. However, our analyses demonstrate the importance of considering farming practices beyond organic and conventional systems to understand apparent idiosyncratic responses. Tillage intensity was one of the main differences that discriminated between organic and conventional systems, as organic farmers cannot use synthetic herbicides to control weeds ^32^. Our analyses show that organic farming benefits springtail abundance in the topsoil through higher tillage intensity, which may increase food availability and limit soil compaction ^19,33,34^. The positive effect of organic farming on spider abundance confirms results from other studies ^20,28,35^. Our results indicate that this positive effect is not driven by tillage intensity or any other covariable related to farming practices or soil conditions (see Suppl. Mat. Figure S10) but suggest that spiders may have benefited from the higher prey availability resulting from organic farming (as found for springtails) ^33,36^. However, not all organisms benefited from higher trophic resource availability under organic farming, as pollinator abundance and soil microbial biomass decreased in organic fields compared to conventional fields. This may appear contradictory to what has been demonstrated in the literature as, on average, both pollinators and soil microbial biomass have been found to benefit from organic farming compared to conventional farming ^13,15^. Again, our analyses provide insights into the key role of farming practices in explaining these effects, as both pollinator abundance and soil microbial biomass were negatively affected by the higher tillage intensity found in organic fields. Higher tillage intensity strongly limits flower availability and the emergence of ground-nesting wild bees ^37,38^. Similarly, higher tillage intensity is known to create less favorable environmental conditions that reduce soil microbial biomass compared to that under reduced or no-tillage systems ^39,40^. Finally, tillage intensity and clay content in the soil strongly limited the abundance and richness of ground beetles independently of the type of farming system, showing that the practices composing farming systems are an important aspect to consider to understand the overall effect of the type of farming system. Such effects occur because the life cycle of ground beetles is strongly related to soil conditions ^41^, and a reduction of the abundance of ground beetles by half due to tillage has already been recorded ^42^.

We expected that the proportion of organic farming in the landscape would be a strong driver of above- and belowground communities. However, we found that organic farming in the landscape had little effect on the abundance or taxonomic richness of the above- and belowground communities, indicating that the scale of effect of organic farming was mainly the field scale for the taxonomic groups we considered. We therefore did not validate our hypothesis stating that the scale of effect of organic farming would be driven by the trophic level considered and its average dispersal ability. For instance, we could have expected that spiders would benefit from a higher proportion of organic farming in the landscape as they strongly benefit from organic farming at the field scale and they have relatively high dispersal abilities ^28,26^. The fact that organic farming in the landscape had little effect on multitrophic biodiversity might have result from very limited spillover between fields resulting from negative impacts of other farming practices in the landscape, such as pesticide use ^43^, the spatial arrangement of organic farming in interaction with other aspects of landscape structure ^44^ or a gradient of organic farming proportions (i.e., 0–24% in a 1 km radius) that is not sufficient to detect effects on above- and belowground communities ^29,45^. Only ground beetles responded to organic farming in the landscape in terms of both abundance and taxonomic richness. Ground beetles were negatively affected by an increase in the area of organic farming. This suggests upscaling effects of organic farming at the landscape mediated by higher tillage intensities that limit ground beetle spillover ^46,47^. Landscape complexity characterized by the amount of semi-natural habitats in the landscape was a major driver of changes in the abundances of four of the seven taxonomic groups studied: pollinators, ground beetles, mites and springtails. However, the directions of the effects differed between the above- and belowground communities, as the abundances of pollinators and ground beetles benefited from a higher proportion of semi-natural habitats while the abundances of springtails and mites were reduced by an increasing proportion of semi-natural habitats. Semi-natural habitats play a key role in pollinator and ground beetle communities because they provide food sources, overwintering sites and refuges from disturbance ^17,48^. Maintaining such habitats in vineyard landscapes is therefore essential for these communities. In contrast, negative effects of the proportion of semi-natural habitats on springtail and mite abundances have already been reported ^49,50^, suggesting physical barriers to passive dispersal ^51^ or greater top-down control by their predators in more complex landscapes ^33^. Moreover, a significant effect of the interaction between local farming system and the proportion of semi-natural habitats appeared in five of seven models explaining the abundance of above- and belowground communities, indicating that landscape context modulates the local effect of farming systems, as demonstrated in other studies ^52,53^. A very interesting result of our study is that, independently of the type of farming system, the use of insecticides (either organic or synthetic) decreased the abundances of pollinators, ground beetles and springtails as well as the taxonomic richness of pollinators. Insecticides are known to have both lethal and sublethal effects on bees and natural enemies ^54^, by affecting reproductive success, immunity, mobility or foraging ability^54^. Our study clearly indicates that such negative effects are detectable in the field, as we found decreases of 32.9% and 20.3% in pollinator abundance and taxonomic richness as well as decreases of 80.7% and 73.3% in the abundances of ground beetles and springtails, respectively, for a treatment intensity ranging from 0 (no insecticide) to 4 (4 full doses of insecticide applied per field). We were not able to examine the effect of fertilizers use on above- and belowground communities as very few farmers used fertilizers. However, investigating how the type and the amount of fertilizers affect multiple trophic groups through bottom-up effects would be of major interest. Our analyses also suggest that farming practices may impact communities on much longer temporal scales than the field season. Indeed, copper accumulates in the topsoil and is strongly affected by the historical use of copper-based treatments ^18^. Our results show negative effects of copper content in the soil on the abundances of springtails and soil microarthropods, indicating that soils with higher concentrations of copper had detrimental effects on belowground communities independent of the type of farming system ^55^. Copper is used in both organic and conventional systems because of its fungicidal properties and therefore affects microbial communities in soil as well as other organisms involved in nutrient cycling ^56^. Considering how temporal dynamics in farming practices affect multiple communities is therefore of crucial importance for designing agricultural landscapes that buffer against biodiversity declines ^57^.

It has been recently argued that simple comparisons between broad categories of farming systems could hinder a mechanistic understanding of the socioecological impacts of different forms of agriculture ^24^. To our knowledge, our study provides the first empirical test of this idea and demonstrate the added value of analyzing both aspects to stimulate the development of innovative cropping systems. Our modelling approach considering two different sets of explanatory variables, one at the farming system scale (model 1) and one considering individual farming practices (model 2) for each community, provides important and complementary information to understand the relationships between agriculture and farmland biodiversity. Interestingly, and despite the fact that both models were always informative for community-abundance models (Table S1), the relative quality of model 1 and 2 provides information about the responses of each group to farming practices. Models considering the type of farming systems (model 1) were always more informative than models considering individual farming practices (model 2) for the abundances of pollinators, ground beetles, spiders, springtails and microbial biomass (Table S1). This indicates that there might be other key aspects discriminating organic and conventional farming systems that are important for these communities that are not captured by individual farming practices considered in our study. However, models considering individual farming practices were better than models considering the type of farming system for abundances of mites and soil microarthropods, suggesting that specific farming practices independently of the certification scheme are more important for these communities (Table S1).

Our study carefully analyzing how farming practices at different spatial scales affect multiple components of biodiversity represents a major step forward in understanding the relationships between farming practices and biodiversity in agricultural landscapes. The field scale was found to be the most important scale of effect of farming practices across the seven taxonomic groups considered, and organic farming at this scale mainly affected abundance, not taxonomic richness. The low explanatory power of models explaining taxonomic richness of multiple communities by farming practices is not surprising as a previous study in the same study region reported that taxonomic richness or arthropods do not differ significantly between organic farming and conventional fields ^58^. Of course, other environmental variables such as abiotic factors not included in our analyses may have impacted taxonomic richness or community composition of the different communities. However, our study is largely focused on arthropods and it would be interesting to include other species with different life-cycles and habitat domains such as plants, birds or bats in such analyses. Our results suggest that independently of the type farming system, specific features of agricultural practices, such as tillage intensity and pesticide use, drive the contrasted responses of the abundance of the different taxonomic groups. Future research could explore how trophic interactions across functional groups contribute to explain the effects of agricultural management on biodiversity.

Moreover, while our results are based on highly replicated sampling points in space, we acknowledge that investigating legacy effects between farming practices and biodiversity dynamics using repeated measures over time would be of major interest. Our study provides key information for further designing farming systems that minimize negative impacts on multitrophic biodiversity and highlight key agronomic issues related to organic and non-organic farming. Decreasing tillage intensity, copper and insecticide use should be of major concern in vineyard landscapes if we are to conciliate grape production and biodiversity conservation. Such agroecological pathway implies finding technical solutions for farmers to reduce tillage and pesticide use while limiting competition for nutrients and pest pressure. Future research aimed at quantifying the consequences of the detected changes in community abundances on agroecosystem functioning is now of major importance to fully anticipate the effect of expanding agroecological practices at the global scale.

## Material and methods

### Experimental design and field characteristics

Our study sites were located in a vineyard-dominated region in southwestern France (44°48’N, 0°14’W). Our study design consisted of 20 pairs of organic and conventional vineyards (40 fields). The vineyards were selected in order to obtain pairs distributed along two uncorrelated (Pearson correlation = -0.33, p-value > 0.05) landscape gradients: a gradient of proportion of organic farming (ranging from 0.1% to 24.2%) and a gradient of proportion of semi-natural habitats (ranging from 0.4% to 75.1% and composed of semi-natural forests (65%) and open habitats (35%) such as meadows and shrublands) in a 1 km radius (Figure S12 and S13). Such an experimental design makes it possible to disentangle the relative effects of local farming practices from the proportion of semi-natural habitats and farming practices at the landscape scale. Landscape variables were calculated using QGIS 2.18.1 (QGIS Development Team 2016).

Information about the farming practices of the 40 fields was collected from farmers using a structured interview. We collected data on pesticide use (type of molecule, quantity applied and area treated), which could be organic or not, and soil tillage intensity. To quantify the level of pesticide use, we calculated the treatment frequency index (TFI), which corresponds to the number of recommended doses used per hectare, for the different groups of pesticides (insecticide, fungicide and herbicide) used in organic and conventional farming systems. The TFI includes all type of synthetic or non-synthetic (eg, copper) products applied by farmers. The TFI was respectively calculated for insecticides, fungicides and herbicides as the sum of the ratios between the applied and recommended doses for each application ^59^ (the TFI was not weighted by area treated as the total area of each field was treated for each applications in all fields). This indicator therefore quantifies the number of recommended doses applied per field for each pesticide groups. Soil tillage intensity (Tillage) was calculated as the ratio between the surface under tillage and the total surface of the fields, considering both row and interrow management. We used this approach because the proportion of soil area under tillage varies between fields and because winegrowers use different types of soil tillage under the vine row and between vine rows (i.e., interrows), which can include tilling or mowing every other row.

In addition, we characterized soil texture using the proportion of clay in the topsoil (0–15 cm) and measured the amount of copper in the soil (EDTA Copper, ISO 22066 norm) by mixing 9 subsamples extracted using soil cores (5 cm in diameter) at 15 m intervals in all rows and interrows (tilled or not) from the topsoil (0–15 cm).

### Aboveground communities

Pollinators were sampled between April and August 2018 using colored pan traps and sweep netting. From April to May, pollinators were collected using pan traps on three sampling dates, with two sampling points per field and per date. The two sampling points were located in grassy interrows 15 m apart and were active for 48 h. Each sampling point was composed of two sets of three colored pan traps (blue, yellow and white), one set localized at ground level and the other set localized at vegetation level (60 cm from the ground level)^60,61^. Each colored trap was made of 500 mL plastic bowls, filled with soapy water. From July to August, two sweep netting sessions, with a sweep net of 35 cm in diameter, were performed in all fields. For each session, sweep netting was conducted along two 30 m transects, one in a grassy interrow and one in a tilled interrow, spaced by 15 m and starting at 15 m from the edge. Each transect was sampled using one sweep per footstep. For each field and session, we repeated this operation twice during the same day: in the morning (before 12 p.m.) and in the afternoon (after 2 p.m.). Samplings occurred on dry and sunny days with low wind speeds. All collected individuals were stored in 70% ethanol, and individuals were identified to the lowest possible taxonomic resolution. Only wild pollinators (bees, bumble bees and hoverflies) were considered for the analyses, while honey bees (*Apis mellifera*) were removed from the analyses to avoid bias due to the presence of beehives around plots. To calculate taxonomic richness, we used taxonomic units based on the lowest level of identification bees, bumblebees and hoverflies. For bees of the genus *Lasioglossum*, due to the difficulty of identification at the species level, we considered subgenera, and based on the strength of the distal veins of the forewing, we divided the bees into two groups under the same subgenus.

Predators were sampled on three different dates in June, July and October 2018 with three pitfall traps for each field and date. Pitfall traps were made of 750 mL plastic cups with 11.5 cm diameter. On each date, the pitfall traps were placed along a transect under a vine row starting 15 m from the field border and were located 15 m from each other. The transect was located towards the middle of the field. The pitfall traps were half filled with soapy water and were left open for 10 days. Spiders and ground beetles were collected and stored in 70% ethanol. Individuals were identified to the lowest possible taxonomic resolution: the species level for all carabids; for spiders, 11% to the family level (e.g., Lycosidae), 12% to the genus level (e.g., *Pardosa* sp*.*) and the rest to the species level.

### Belowground communities

Soil arthropods were collected from the topsoil (0–15 cm) in October 2018. In each of the 40 fields, 500 mL of soil was constituted by mixing 9 subsamples extracted using soil cores (5 cm in diameter) spaced at 15 m intervals in all rows and interrows (tilled or not). Soil arthropods were then directly extracted using a Berlèse-Tullgren extractor for five days ^62^ (ISO 23611–2:2006 norm), with a light and associated temperature gradient over the soil core (48 h without light and 72 h days with light), which was crumbled into a 2 mm plastic sieve suspended over a collecting vessel containing 70% alcohol. All arthropods collected were counted and identified to the order or family level, and springtails were identified to the species level. We divided soil arthropods into three groups: springtails, mites and other microarthropods including small spiders, ants, ground beetle larvae, symphylans, pauropods, chilopods, isopods, diplopods, diplurans and proturans. Soil microbial biomass was collected from the topsoil (0–15 cm) in November 2018. In each of the 40 fields, 500 mL of soil was constituted by mixing 9 subsamples extracted using soil cores (5 cm in diameter) spaced at 15 m intervals in all rows and interrows (tilled or not). We quantified the microbial biomass by fumigation following the ISO 14240–2 norm.

### Statistical analyses

**Figure 2:**
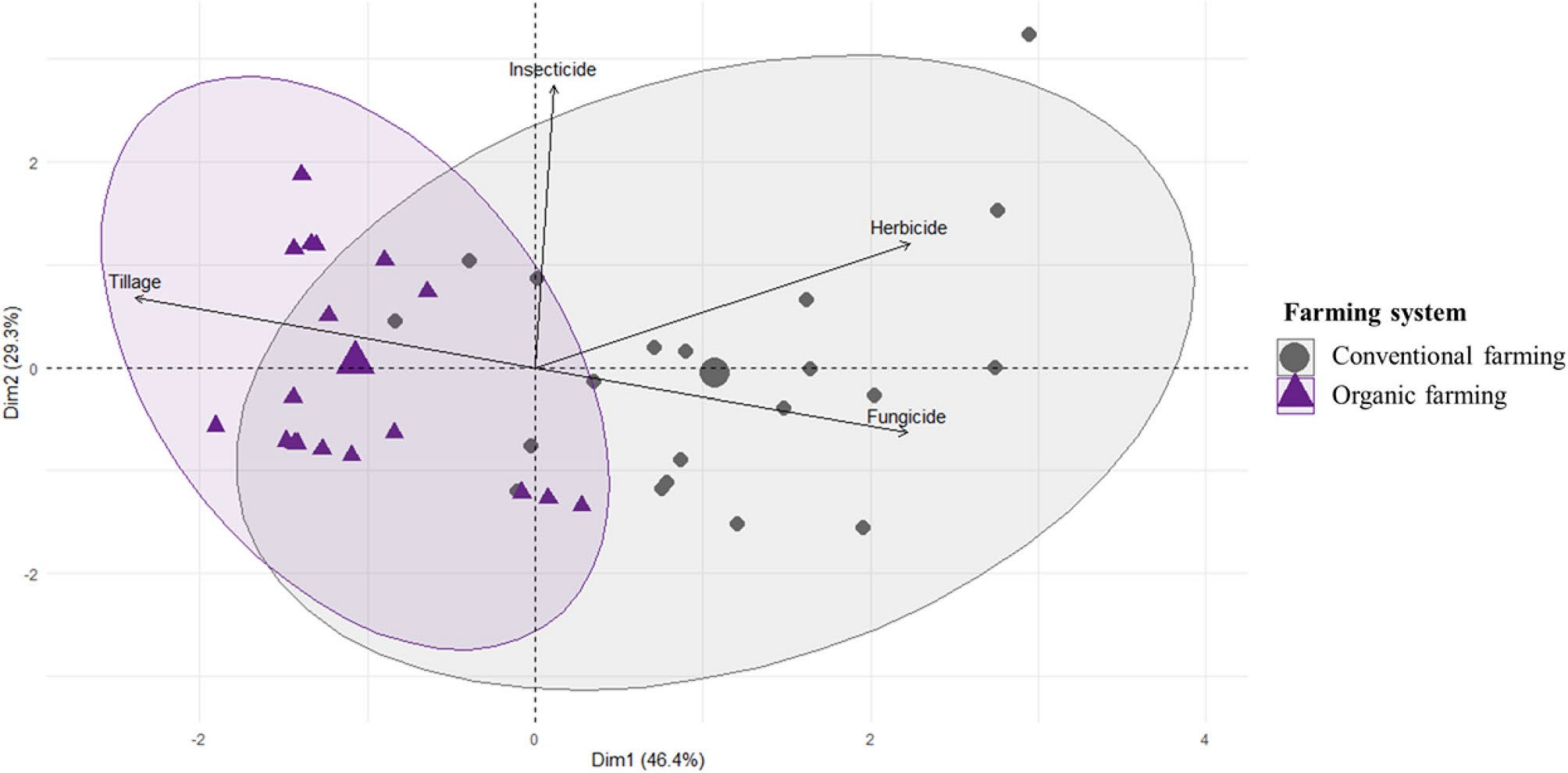
Principal component analysis (PCA) of the four variables used to characterize profiles of agricultural practices in our study. Purple triangles represent plots under organic systems, and grey circles, systems under conventional farming. The largest triangles and circles represent centroids of the ellipses characterizing organic and conventional farming systems, respectively. Pearson correlation matrix between variables are provided in Suppl. Mat. Fig S13. This figure was made using R version 4.0.3 (https://www.R-project.org/).

In order to select relevant and uncorrelated explanatory variables describing differences between organic and conventional systems, we first performed a principal component analysis (PCA) of farming practices based on the collected information. Tillage intensity as well as levels of insecticide, herbicide and fungicide use were included in the PCA (scaled variables). We did not include the amount and type of fertilizers as very few farmers used fertilizers. The PCA identified two main independent axes of farming practices that jointly explained more than 75% of the total variance, with axes 1 and 2 explaining 46.4% and 29.3% of the total variance, respectively (Fig. 2). The first axis discriminated organic from conventional farming systems, with organic farming systems associated with higher tillage intensity and conventional farming systems associated with higher levels of herbicide and fungicide use (Fig. 2 and Table S1). The second axis was independent of organic and conventional systems and was associated with the level of insecticide use (Fig. 2). To avoid redundant information and collinearity in subsequent analyses, we selected soil tillage intensity and insecticide use as two independent variables describing differences in farming practices between fields.

Second, we examined how local farming practices and landscape context affect above- and belowground communities by using generalized linear mixed models (GLMMs) and a multimodel inference approach ^63^. We constructed two models (model 1 and model 2) with different sets of explanatory variables for abundance and taxonomic richness of each community considered (e.g., abundance and taxonomic richness of pollinators). Model 1 included a set of fixed effects, namely, local farming system (organic or conventional) and proportions of semi-natural habitats and organic farming in the surrounding landscape, as well as 3 independent variables, namely, insecticide use intensity and soil copper and clay contents. These three local covariables were included in the full model as they were not associated with the type of farming system. We also added interaction terms between the local farming system and the proportion of semi-natural habitats and between the local farming system and the proportion of organic farming to test for potential modulation of the local effect of farming practices by landscape context. Model 2 had the same structure except that the local farming system was replaced by tillage intensity to examine if potential differences between organic and conventional farming systems were indeed related to differences in soil tillage intensity between these two systems. We included tillage intensity in the model based on the outputs of the PCA of farming practices, which showed that tillage intensity as well as fungicide use intensity was highly correlated with the first axis (axis 1) and nicely discriminated organic from conventional systems.

We fitted generalized linear mixed models (GLMMs) with a Gaussian distribution to explain the taxonomic richness of pollinators, ground beetles, springtails and soil microarthropods as they were normally distributed. We used the Poisson distribution to explain the abundances of pollinators, ground beetles, springtails, mites, and soil microarthropods, microbial biomass and the taxonomic richness of spiders. Finally, we used a negative binomial distribution to explain the abundance of spiders. All the models were fitted with “site” as a random term (one organic and one conventional plot in the same site) to account for the experimental design.

For each set of models, all the possible candidate models were ranked using the Akaike information criterion with correction for small sample sizes (AICc) and models with a ΔAICc < 2 were retained among the set of best models for inference^63,64^ (see outputs of models in Suppl. Mat. Table S1). Such set of best models was then used to estimate the mean effects and confidence intervals of each explanatory variable using model averaging and the full averaging procedure of models. For models fitted with a Gaussian distribution, multimodel inference was performed with the maximum likelihood (ML) ratio, and selected models were refitted using restricted maximum likelihood (REML) to obtain standardized estimates and p-values ^63^. For models with Poisson and negative binomial distributions, multimodel inference was performed under REML. Multimodel inference was performed using confidence intervals on the full average model using the MuMIn package ^65^. All analyses were performed using R (R Core Team)^66^. Correlation matrix between all explanatory variables are provided in the Supplementary Material (Figure S13). The residuals of the models were checked for normality and homoscedasticity using the “DHARMa” pacvkage ^67^. Collinearity between explanatory variables was assessed using the variance inflation factor (all VIFs were lower than 2). All continuous explanatory variables were scaled by the mean and standard deviation. GLMMs were fitted using the “lme4” package ^68^.

We evaluated the relative importance of the predictors in explaining the abundance and richness of each community by calculating the percentage of variance they explained based on the ratio between the absolute values of the standardized regression coefficient and the sum of all standardized regression coefficients of predictors for each model ^57^.

### Ethical approval and informed consent

Informed consent was obtained from all farmers. Information about their farming practices were carried out in accordance with relevant guidelines and regulations including the General Data Protection Regulation 2016/679 (GDPR). All experimental protocols were approved by INRAE.

## Supplementary Information

Supplementary Information.

## Data Availability

The datasets used in the study will be available from DRYAD repository after acceptance of the paper.
