# Supplementary material for: Multi-community effects of organic and conventional farming practices in vineyards
Source: Sci Rep. 2021 Jun 7;11:11979. doi: 10.1038/s41598-021-91095-5 (PMC8184894; doi:10.1038/s41598-021-91095-5)
Supplement: Supplementary file 1 — Supplementary Information. [file 41598_2021_91095_MOESM1_ESM.docx]

**Supporting information**

Multi-community effects of organic and conventional farming practices in vineyards

Noémie Ostandie^a^, Brice Giffard^a^, Olivier Bonnard^a^, Benjamin Joubard^a^, Sylvie Richart-Cervera^a^, Denis Thiéry^a^, Adrien Rusch^a^

^a^ INRAE, ISVV, UMR1065 Santé et Agroécologie du Vignoble, F-33883 Villenave d’Ornon, France

**Communities description***.* For the aboveground communities, we collected 4261 pollinators, with 3 950 bees (5 families, 15 genera), 293 hoverflies (1 family, 15 genera) and 18 bumblebees (1 family, 1 genus). Bees were dominated by two genera, *Lasioglossum* (70% of the total number of bees) and *Andrena* (28%), and hoverflies were dominated by the two genera *Melanostoma* (49%) and *Sphaerophoria* (16%). For predators, we collected 1023 ground beetles (21 genera) and 7 228 spiders (17 families, 65 genera). Ground beetles were dominated by three genera, *Harpalus* (49%), *Pseudophonus* (25%) and *Nebria* (10%), and spiders were dominated by the genus *Pardosa* (68%).

For the belowground communities, we collected 4101 microarthropods, with 796 springtails (28 genera, 52 taxonomic units), 2819 mites and 486 other microarthropods. Springtails were dominated by three genera, *Cryptopygus* (36%), *Sphaeridia* (10%) and *Folsomides* (10%). Microarthropods were dominated by juveniles of small spiders (39%) and ants (36%).

The microbial biomass in plots ranged between 44 mg/kg and 166 mg/kg of dry matter (mean ± SE= 105 ± 44 mg/kg).

**Results on taxonomic richness**

**Effects of organic farming at the field and landscape levels.** Organic farming at the field scale was only retained in the best models explaining the taxonomic richness of ground beetles and spiders and was found to be significant only for spiders (Suppl. Mat Figure S9). Compared with conventional farming, organic farming increased the taxonomic richness of spiders by 23.2% (Suppl. Mat Figure S1). Organic farming at the field scale was the most important variable explaining the taxonomic richness of spiders, as it accounted for 76% of the explained variance (Suppl. Mat Figure S9).

At the landscape scale, increasing the proportion of organic farming from 0.1% to 24.2% decreased the taxonomic richness of ground beetles by 33% (27% of the explained variance) (Suppl. Mat Figures S3 and S9).

Our analyses revealed that tillage intensity did not explain the observed effects of organic farming at the field scale on taxonomic richness of spiders (model 2 outputs in Suppl. Mat Figure S11). No significant effects of tillage intensity on the taxonomic richness of the above- and belowground communities were found (model 2 outputs in Suppl. Mat Figure S11). The interaction between tillage intensity at the field scale and the proportion of organic farming did not account for the variance explained by all models explaining the taxonomic richness (model 2 outputs in Suppl. Mat Figure S11).

**Effect of semi-natural habitats at the landscape scale.** The proportion of semi-natural habitats was selected only in models explaining the taxonomic richness of pollinators (12% of the explained variance), ground beetles (9% of the explained variance) and springtails (27% of the explained variance) (Suppl. Mat Figure S9). No significant effects of the proportion of semi-natural habitats in the landscape on the taxonomic richness of the above- and belowground communities were found.

The interaction between local farming system and the proportion of semi-natural habitats was never selected in any best models explaining the taxonomic richness of the above- and belowground communities, except for that of pollinators.

**Insecticide use**. Independent of the type of farming system, insecticide use intensity was found to decrease the taxonomic richness of pollinators (67% of explained variance) (Figure 1, Suppl. Mat Figures S6 and S9). No significant effects of insecticide use intensity on the taxonomic richness of other above- or belowground communities were found.

**Soil copper and soil texture.** The amount of copper in the soil explained some variance in the taxonomic richness of pollinators (21% of explained variance) and springtails (27% of explained variance), but no significant effects were found. The proportion of clay in the soil explained some of the variance in the taxonomic richness of ground beetles (59% of explained variance), spiders (21% of explained variance) and microarthropods (92% of explained variance). The taxonomic richness of ground beetles was negatively affected by the proportion of clay in the soil, while the taxonomic richness of microarthropods benefited from an increase in clay content (Suppl. Mat Figures S8 and S9).

**Table S1:** Summary of the outputs of the best models (Delta AICc <2) explaining the abundance of multiple groups biodiversity following model 1 (a) and model 2 (b), and to explain the taxonomic richness following model 1 (c) and model 2 (d). The best models summarized in the tables were then used for model averaging (see Figure 1, S9, S10 and S11). Tables are showing the intercepts and estimates for the given explanatory variables as well as the AICc values, marginal and conditional R^2^. “-” indicates that the explanatory variable was not retained in the selected models. Estimates of “farming systems” are reported as the effect of “organic farming” using “conventional systems” as control.


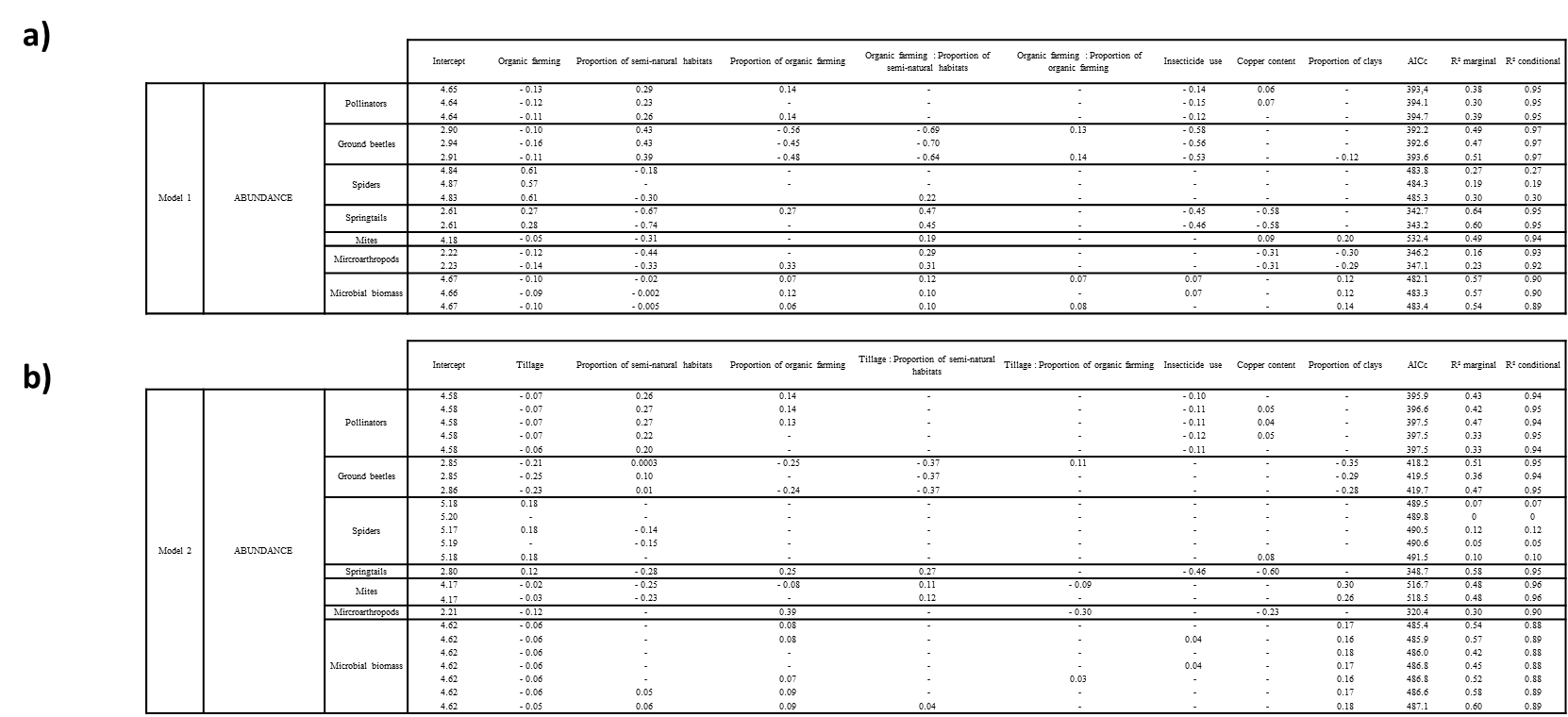


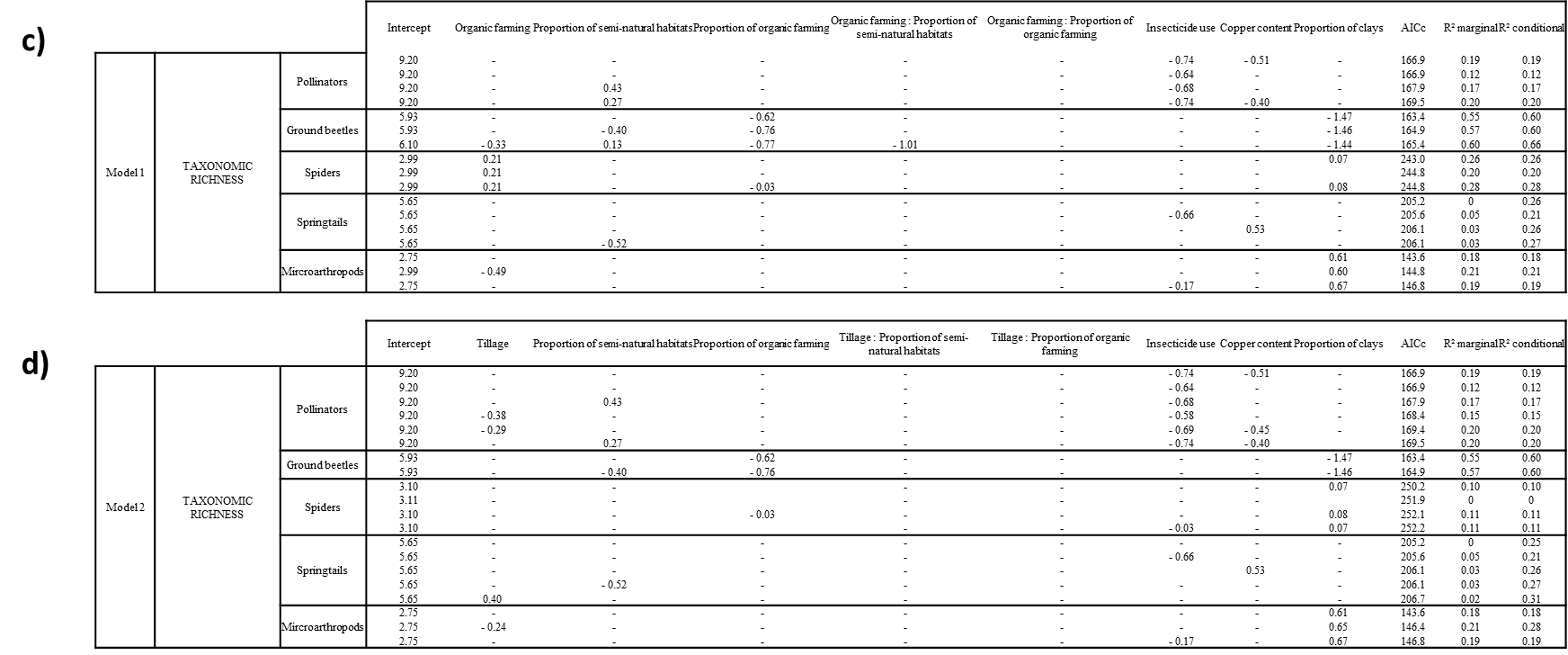


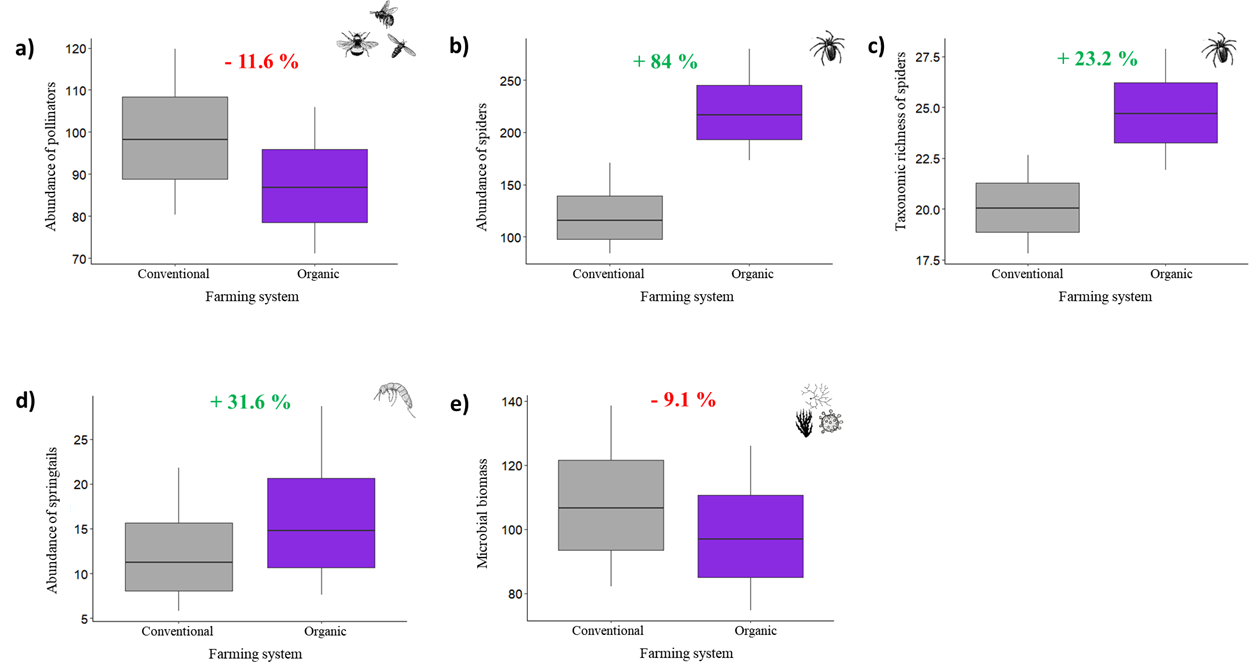


**Figure S1:** Effect of the type of farming system (organic or conventional farming) on the abundances of a) pollinators, b) spiders, and d) springtails; c) the taxonomic richness of spiders; and e) microbial biomass. The proportion of change is calculated on the basis of predictions of the best models obtained from the model averaging procedure. Only significant effects (provided by our models) of the type of farming systems on abundance data for the different groups are represented here. Estimates and confidence intervals for the effect of local farming systems on response variables can be found in Figure 1 and Figure S12.


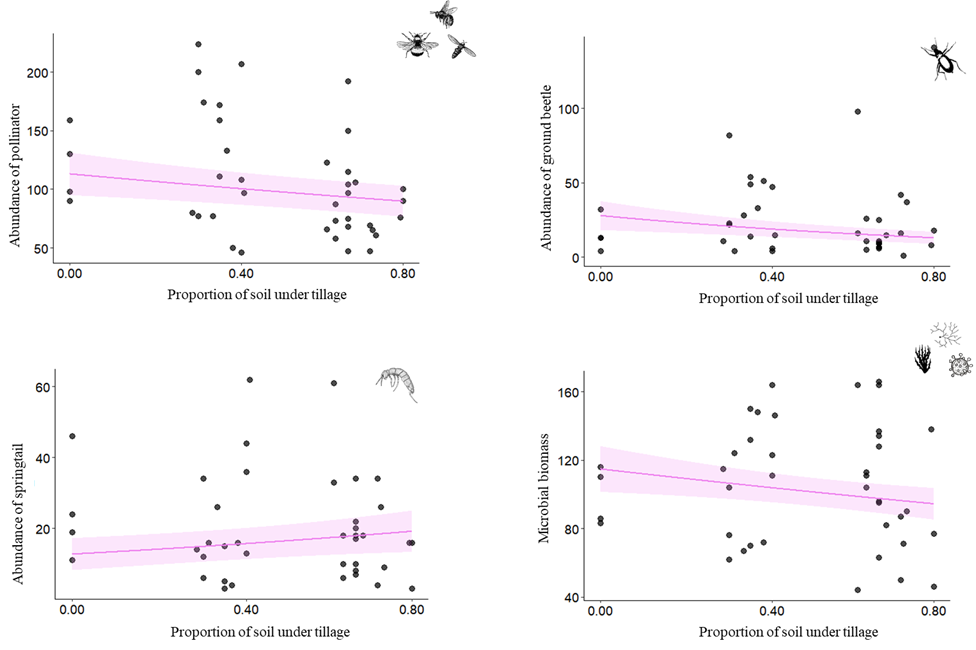


**Figure S2:** Effects of tillage on a) the abundance of pollinators, b) the abundance of ground beetles, c) the abundance of springtails and d) the abundance of soil microarthropods. Black dots represent raw data of abundance or taxonomic richness. Lines show predictions from the models, and the pink parts are confidence intervals.

**
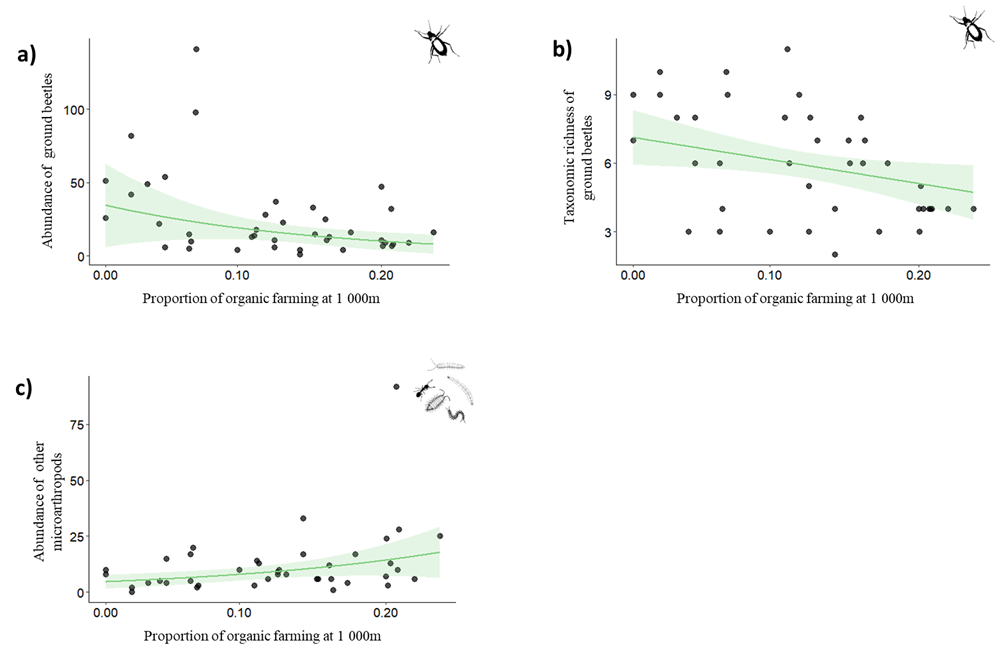
**

**Figure S3:** Effects of the proportion of organic farming within a 1 km radius on a) the abundance and b) the taxonomic richness of ground beetles and c) the abundance of soil microarthropods. Black dots represent raw data of abundance or taxonomic richness. Lines show predictions from the models, and the light green parts are the confidence intervals. Only the results from the plots under organic conditions are shown when farming system was selected in the final model.


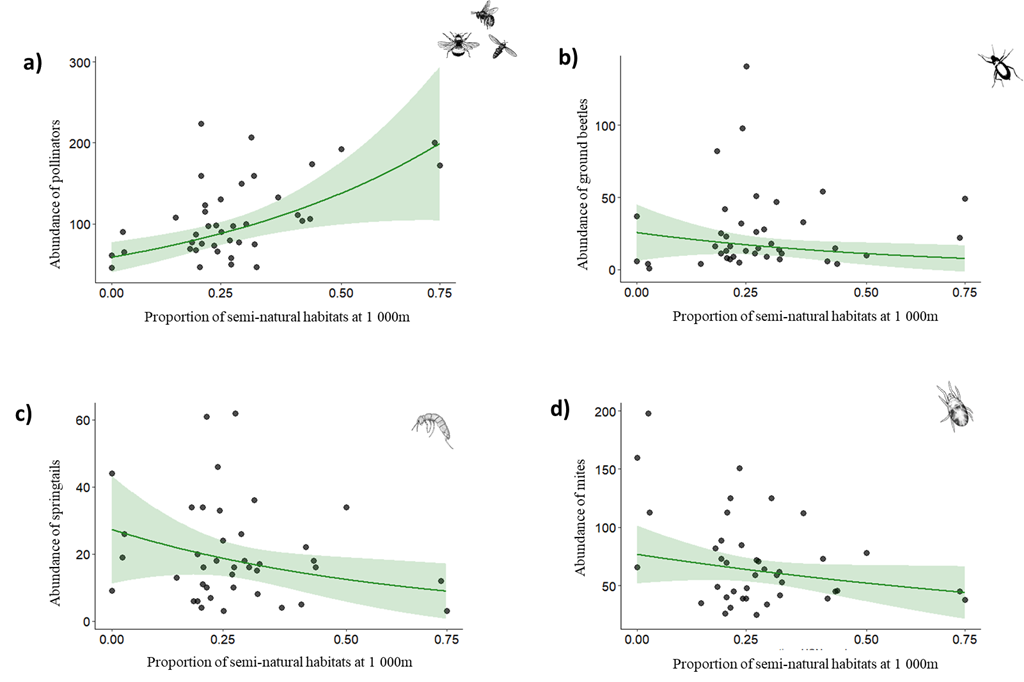


**Figure S4:** Effects of the proportion of semi-natural habitats within a 1 km radius on the abundances of a) pollinators, b) ground beetles, c) springtails and d) mites. Black dots represent the raw data of abundance and taxonomic richness. Dark green lines show the predictions from the model, and the dark green parts are the confidence intervals. Note, that only models for the plots under organic conditions are represented here when farming system was selected in the final model. In order to know how the interactions between the farming system and the proportion of semi-natural habitats is modulating community abundances, see Figure S5 (e.g., for ground beetles).


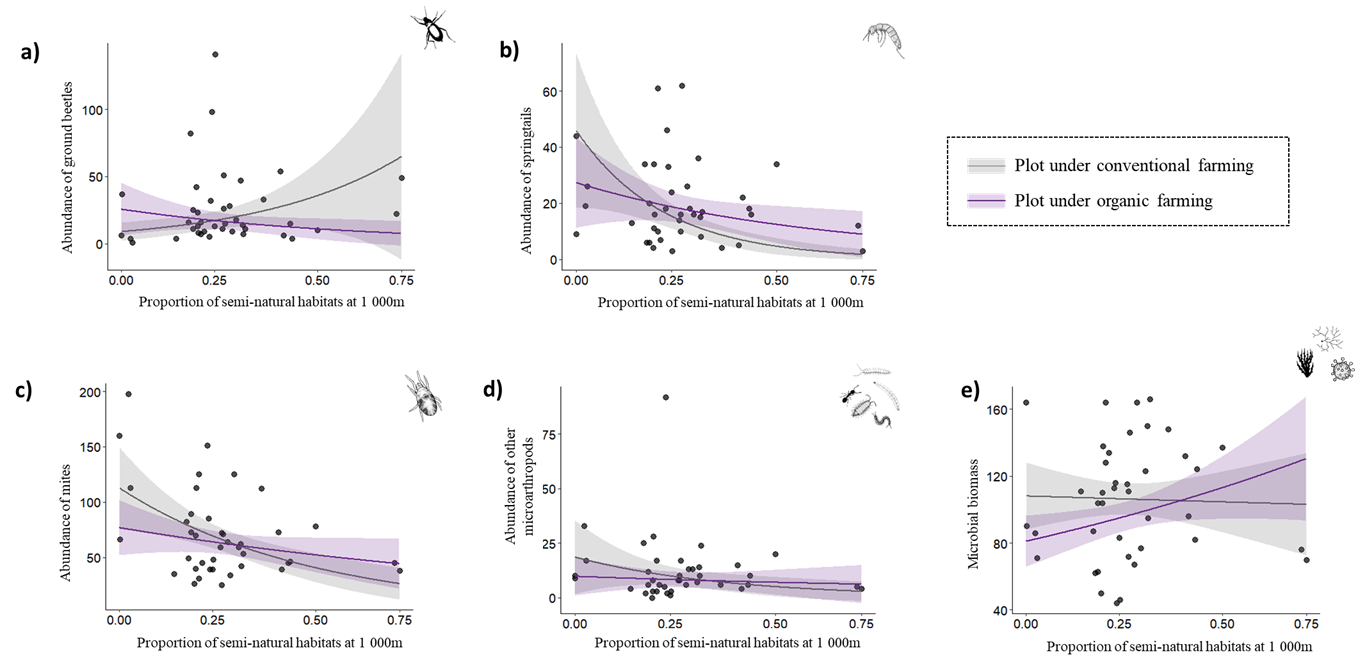


**Figure S5:** Effects of the proportion of semi-natural habitats within a 1 km radius and its interaction with farming system on the abundances of a) ground beetles, b) springtails, c) mites, and d) microarthropods and e) microbial biomass. Black dots represent the raw data for abundance and taxonomic richness. Dark purple and gray lines show predictions from the model and the associated confidence intervals.


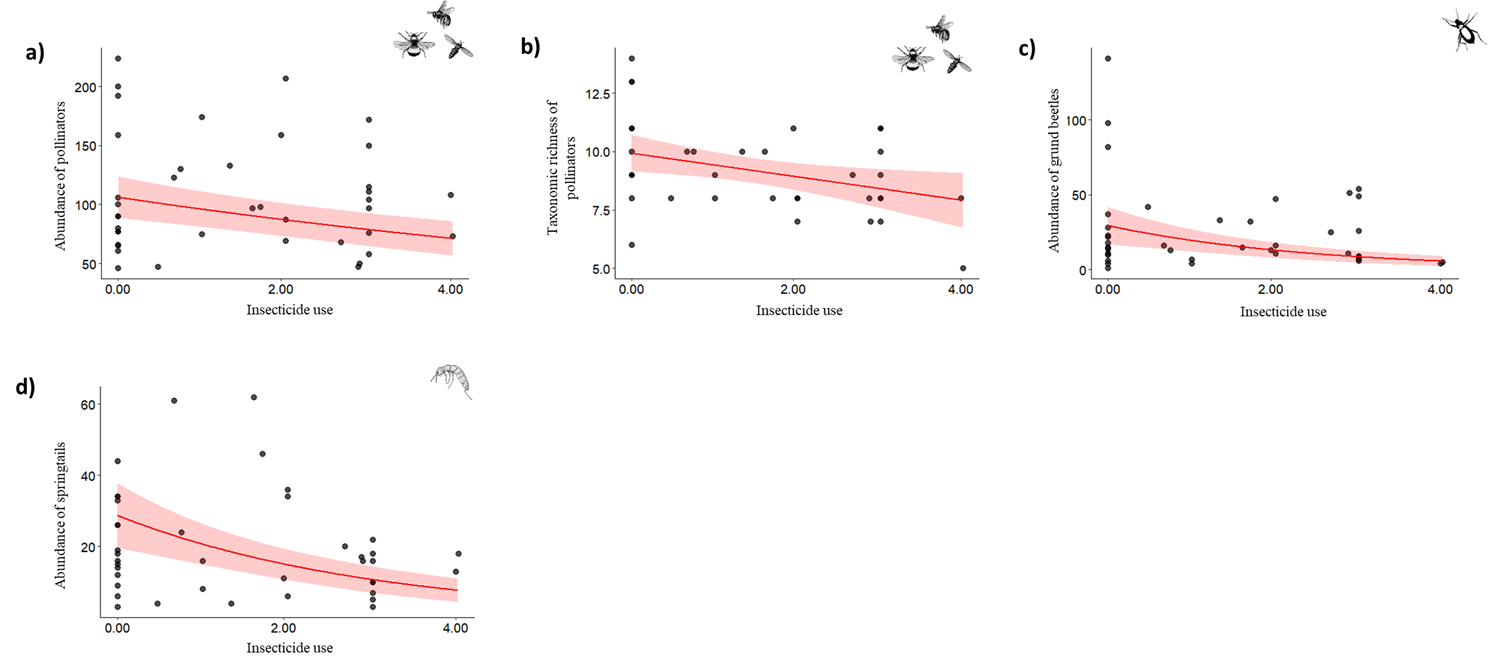


**Figure S6:** Effects of insecticide use on the abundances of a) pollinators, c) ground beetles, and d) springtails as well as on b) the taxonomic richness of pollinators. Black dots represent the raw data of abundance and taxonomic richness. Dark red lines show predictions from the models and the confidence intervals. Only the results from the plots under organic conditions are shown when farming system was selected in the final model.


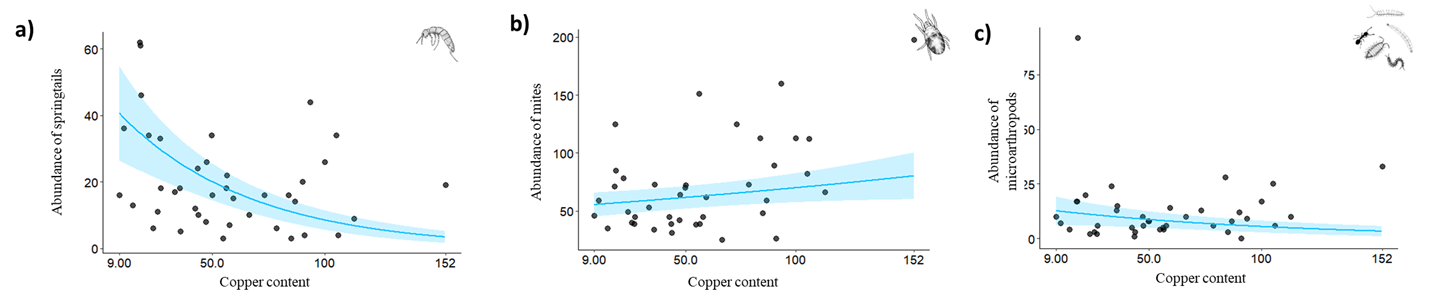


**Figure S7:** Effects of soil copper content on the abundances of a) springtails, b) mites and c) soil microarthropods. Black dots represent the raw data for abundance and taxonomic richness. Light blue lines show predictions from the models and the confidence intervals. Only the results from the plots under organic conditions are shown when farming system was selected in the final model.

**
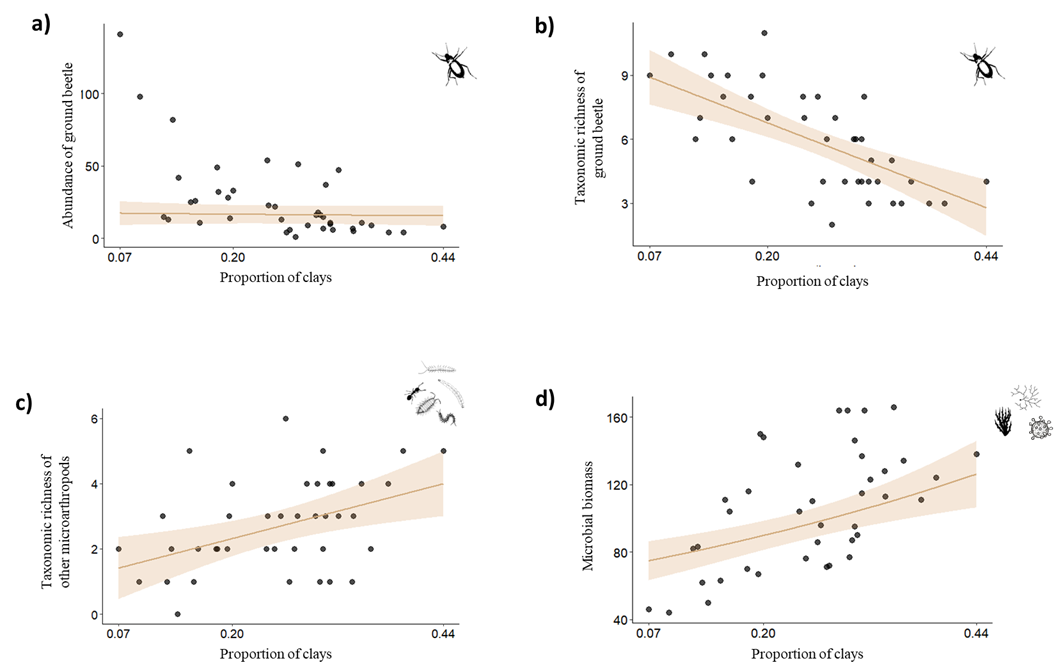
**

**Figure S8:** Effects of the proportion of clay in the soil on the a) abundance and b) taxonomic richness of ground beetles, as well as on the c) taxonomic richness of microarthropods and d) microbial biomass. Black dots represent the raw data for abundance and taxonomic richness. Brown lines show predictions from the models and the confidence intervals. Only the results from the plots under organic conditions are shown when the farming system were selected in the final model.


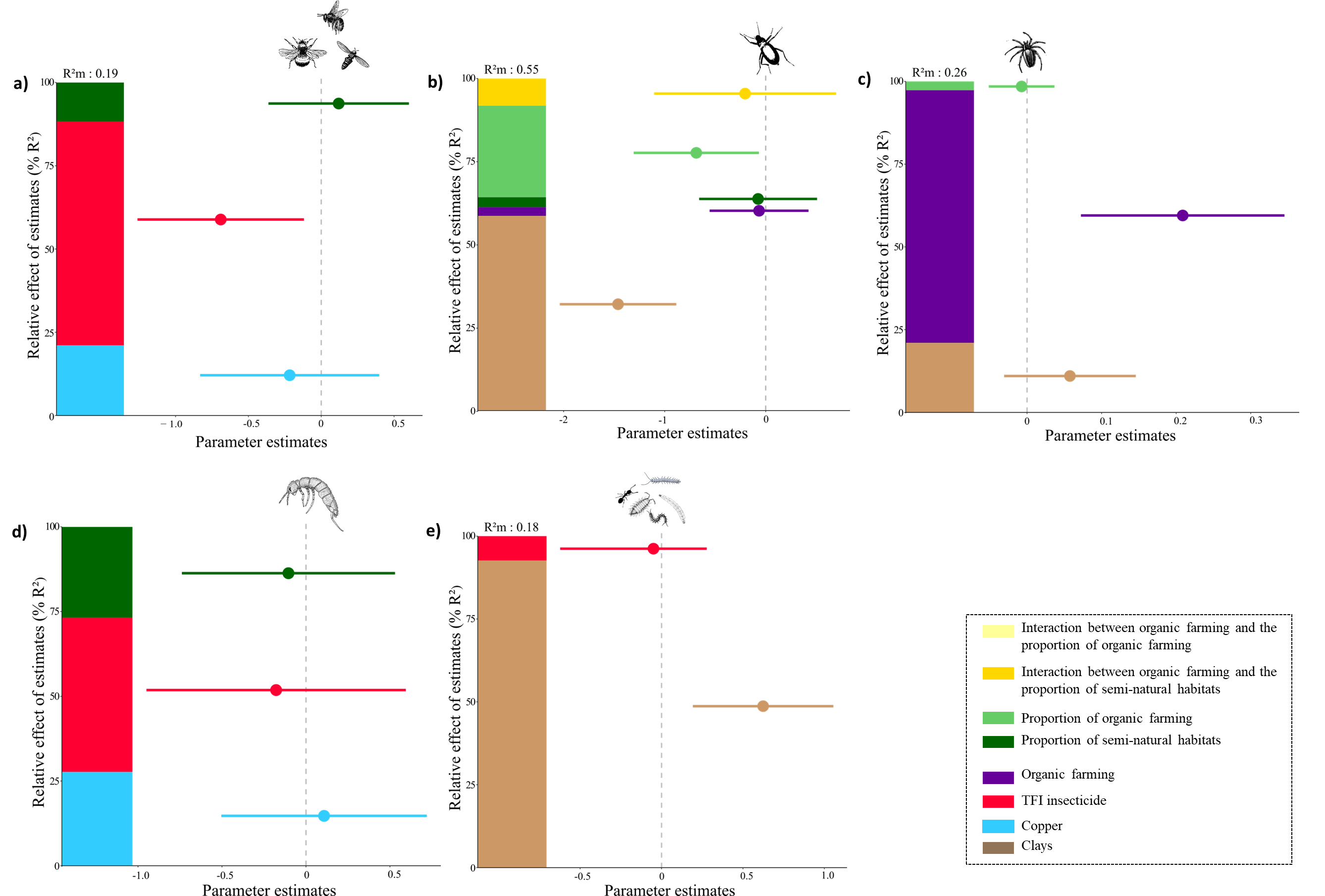


**Figure S9:** Results of the best models explaining the taxonomic richness of communities of a) pollinators, b) ground beetles, c) spiders, d) springtails and e) other soil microarthropods as affected by the type of farming system (organic or conventional), landscape context, farming practices independent of farming systems and soil characteristics. Stacked bars show the relative effects of estimates (% R2) for each explanatory variable, which were calculated as the ratios between the parameter estimates and the sum of all parameter estimates based on a model averaging approach applied to model 1 for taxonomic richness. Abscissas represent the average effect of the average model from multimodel inference. Points are estimates of the model, and lines represent confidence intervals. All continuous predictors were scaled to interpret parameter estimates at comparable scales. R²m were not provided for the taxonomic richness of springtails as none of the explanatory variables were significant.


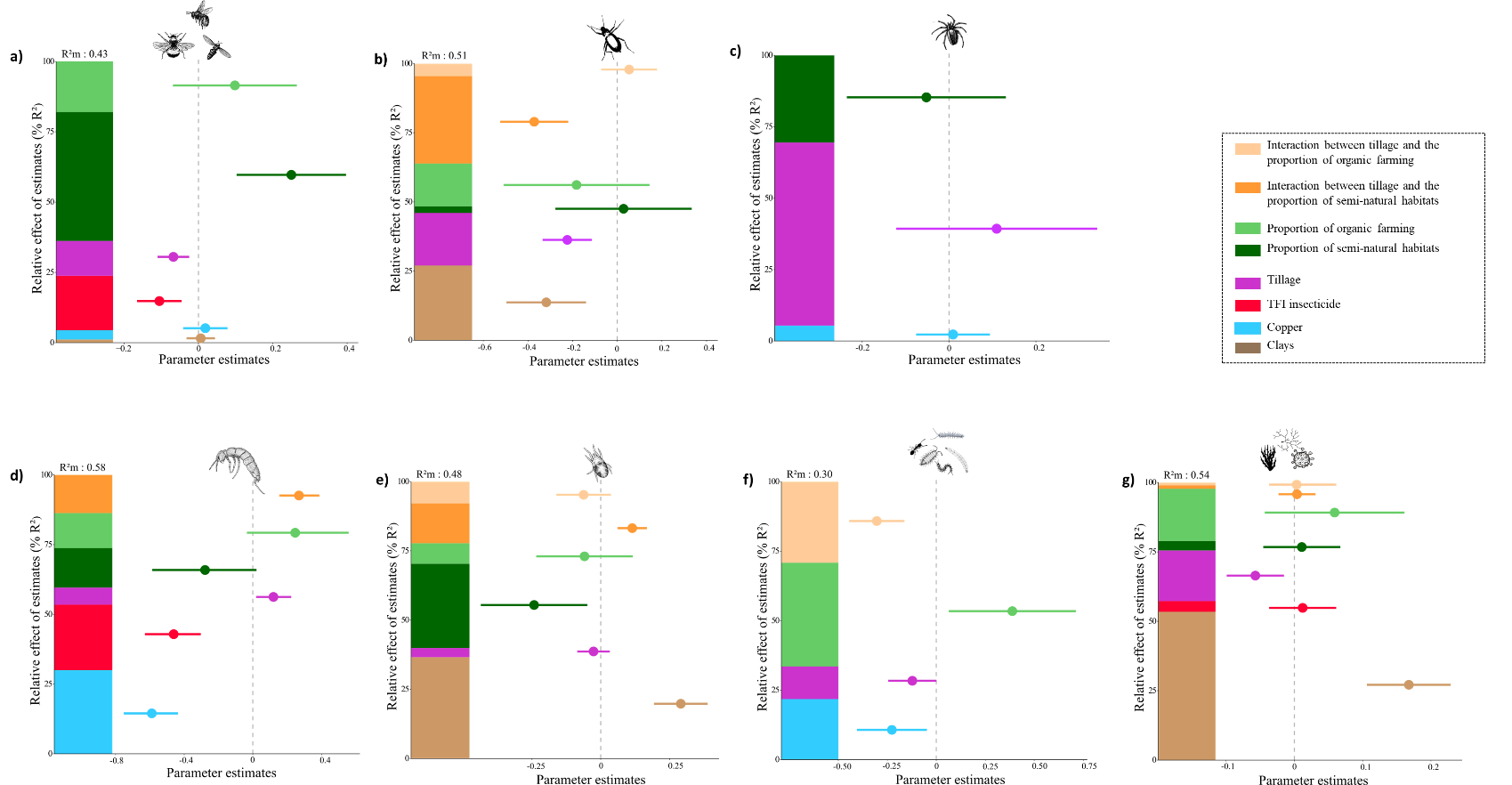


**Figure S10:** Results of the best models of community abundance for a) pollinators, b) ground beetles, c) spiders, d) springtails, e) mites, f) other microarthropods and g) microbial biomass as affected by specific farming practices, landscape context and soil characteristics. Stacked bars show the relative effects of estimates (% R2) for each explanatory variable, which were calculated as the ratios between the parameter estimates and the sum of all parameter estimates based on a model averaging approach applied to model 2. Abscissas represent the average effect of the average model from multimodel inference. Points are estimates of the model, and lines represent confidence intervals. All continuous predictors were scaled to interpret parameter estimates at comparable scales. R²m were not provided for the abundance of spiders as none of the explanatory variables were significant.


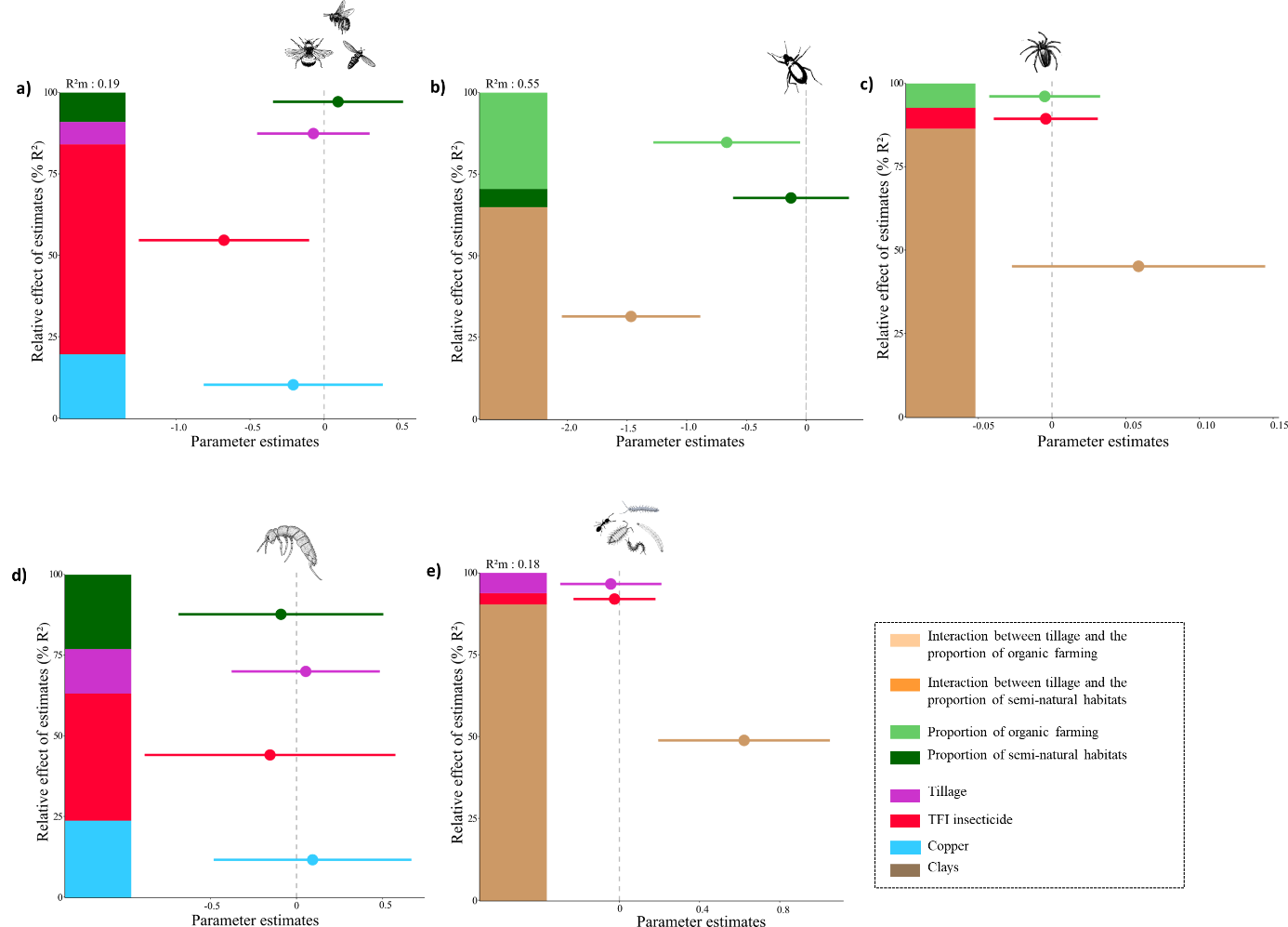


**Figure S11:** Results of the best models explaining the taxonomic richness of communities of a) pollinators, b) ground beetles, c) spiders, d) springtails and e) other microarthropods as affected by specific farming practices, landscape context and soil characteristics. Stacked bars show the relative effects of estimates (% R2) for each explanatory variable, which were calculated as the ratios between the parameter estimates and the sum of all parameter estimates based on a model averaging approach applied to model 2 for taxonomic richness. Abscissas represent the average effect of the average model from multimodel inference. Points are estimates of the model, and lines represent confidence intervals. All continuous predictors were scaled to interpret parameter estimates at comparable scales. R²m were not provided for the abundance of spiders and springtails as none of the explanatory variables were significant.

**
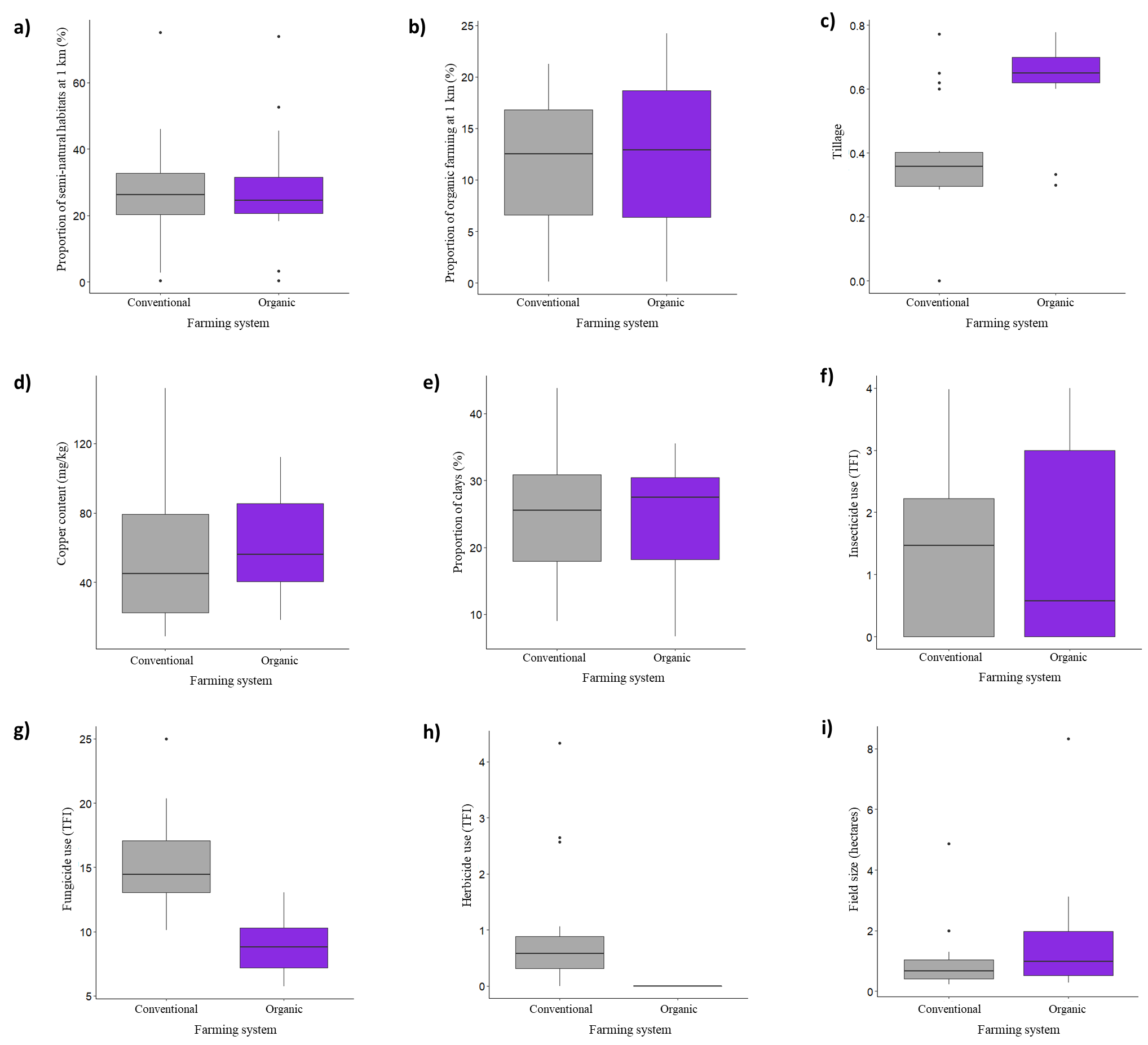
**

**Figure S12:** Box plots representing differences in a) proportion of semi-natural habitats calculated at 1 km of radius, b) proportion of organic farming calculated at 1 km of radius, c) proportion of the field with tillage, d) copper content in soil, e) proportion of clays in the soil, f) insecticide use (TFI), g) fungicide use (TFI), g) herbicide use (TFI) and i) field size between organic and conventional farming systems sampled in the study.


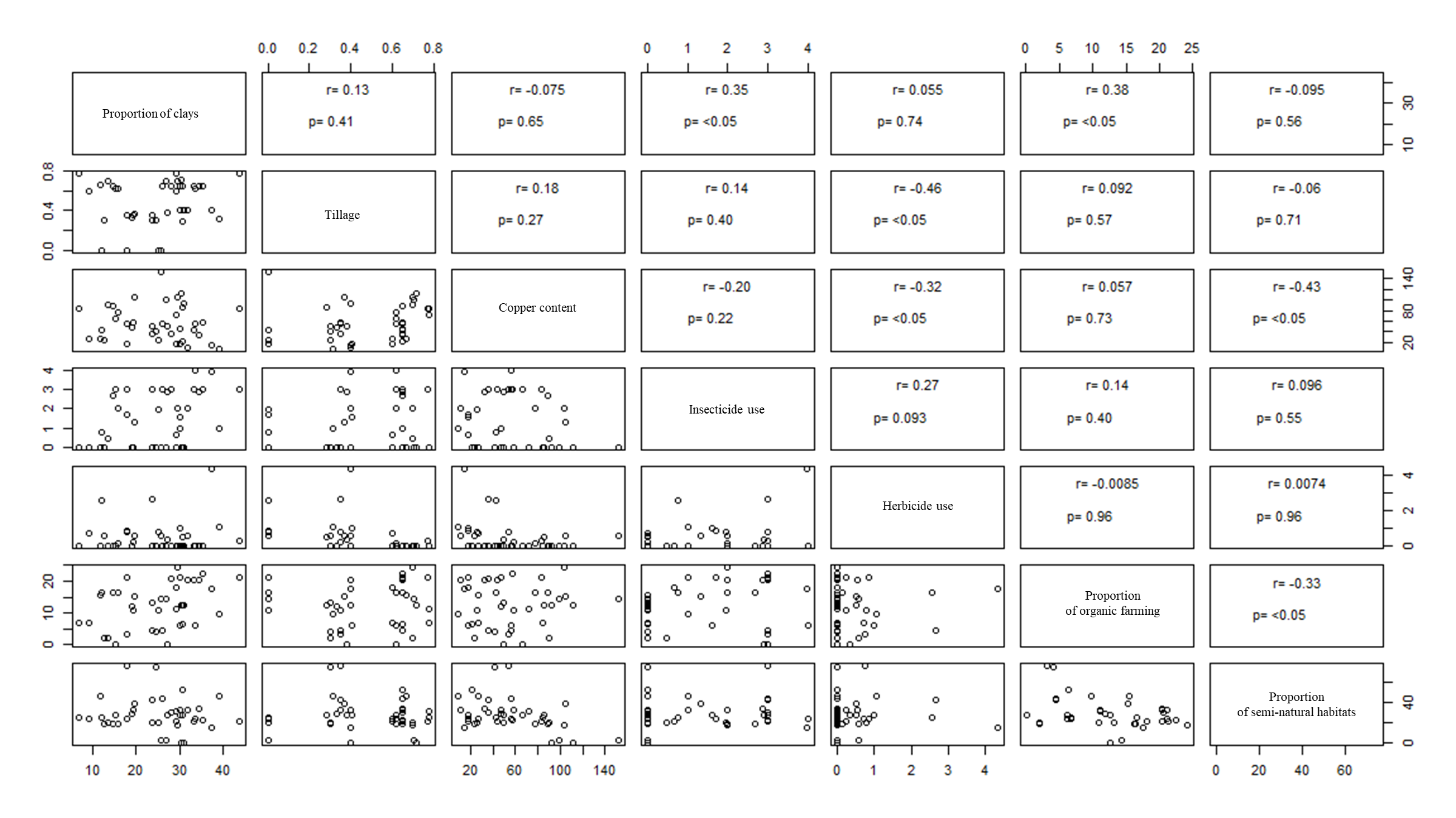


**Figure S13:** Pearson’s correlation matrix between all the explanatory variables. Boxes with text summarize the coefficient of correlation (r) between the two variables and p-value (p) resulting from the Pearson test (p <0.05 reports significant correlation).
